# Prevalence of extensively drug-resistant tuberculosis in a Chinese multidrug-resistant TB cohort after redefinition

**DOI:** 10.1186/s13756-021-00995-8

**Published:** 2021-08-26

**Authors:** Cong Yao, Haiping Guo, Qiang Li, Xuxia Zhang, Yuanyuan Shang, Tongxin Li, Yufeng Wang, Zhongtan Xue, Lu Wang, Liang Li, Yu Pang

**Affiliations:** 1 Department of Bacteriology and Immunology, Beijing Chest Hospital, Capital Medical University/Beijing Tuberculosis and Thoracic Tumor Research Institute, Postal No 9, Beiguan Street, Tongzhou District, Beijing, 101149 People’s Republic of China; 2 Department of Tuberculosis, Beijing Chest Hospital, Capital Medical University/Beijing Tuberculosis and Thoracic Tumor Research Institute, Beijing, 101149 People’s Republic of China; 3 Central Laboratory, Chongqing Public Health Medical Center, Southwest University Public Health Hospital, Chongqing, 400036 People’s Republic of China; 4 Department of Laboratory Quality Control, Innovation Alliance On Tuberculosis Diagnosis and Treatment (Beijing), Beijing, 101149 People’s Republic of China

**Keywords:** *Mycobacterium tuberculosis*, XDR, Susceptibility

## Abstract

**Objectives:**

Recently, the definition of extensively drug-resistant TB (XDR-TB) has been revised. In this study, we conducted a descriptive and retrospective study to determine the prevalence of XDR-TB in a Chinese multidrug-resistant TB (MDR-TB) cohort.

**Methods:**

Broth microdilution method was performed to determine in vitro susceptibilities of *Mycobacterium tuberculosis* (MTB) isolates to (FQs), bedaquiline (BDQ) and linezolid (LZD). The putative drug target genes conferring drug resistance were screened by DNA sequencing.

**Results:**

A total of 425 MDR-TB isolates were included from 13 pilots in China. LZD and BDQ resistance were noted in 30 (7.1%) and 10 (2.4%) isolates. On the basis of latest definitions, 114 (26.8%) were MDR-TB, 282 (66.4%) were pre-XDR-TB, and 29 (6.8%) were XDR-TB. Among 311 FQ-resistant isolates, 265 harbored genetic mutations within QRDRs. The most common mutations were observed at codon 94 of *gyrA*, accounting for 47.2% of FQ-resistant MTB isolates. Only mutations within the *Rv06*78 gene were found to confer BDQ resistance in our cohort, conferring 40.0% of BDQ resistance. For LZD resistance, 53.3% of LZD-resistant isolates carried genetic mutations in *rplC* or 23S rRNA. The most frequent mutation was Cys154Arg in the *rplC* gene. In addition, we recorded two MDR-TB patients with resistance to both BDQ and LZD, of which one patient experienced continuous positive culture of MTB despite inclusion of efficacious moxifloxacin.

**Conclusion:**

Our results demonstrate that the low prevalence of XDR-TB holds great promise for MDR-TB treatment with WHO-endorsed regimens containing BDQ-LZD combination, whereas the high prevalence of FQ-resistance in MDR-TB patients warrants national attention.

**Supplementary Information:**

The online version contains supplementary material available at 10.1186/s13756-021-00995-8.

## Introduction

Tuberculosis, caused by *Mycobacterium tuberculosis* (MTB) complex, continues to be a global public health priority [1, 2]. The World Health Organisation (WHO) estimates that approximately 10.0 million people developed TB and 1.4 million people died from this disease in 2019 [1]. The emergence of multidrug-resistant tuberculosis (MDR-TB; resistant to at least isoniazid and rifampin), with an estimated burden of 78% rifampicin-resistant TB cases worldwide, has jeopardised TB control and subverted the goals of the WHO’s END TB Strategy [1, 3]. Because of inherent resistance to the two most potent anti-TB drugs, MDR-TB treatment requires the use of second-line drugs that are less effective, more toxic, and costlier than first-line regimens [4, 5]; however, the overall rate of treatment success among MDR-TB patients is currently 57% [1]. This unsatisfactory outcome is expected to worsen the epidemic of this severe form of TB. Therefore more efforts are urgently needed for new and effective drugs to improve the chemotherapy of MDR-TB [6].

Over the past decades, several new or re-purposed agents antimicrobial hold promise for MDR-TB treatment, such as bedaquiline (BDQ), linezolid (LZD) and delamanid [6–9]. Recent clinical trials have demonstrated that higher treatment success rates are achievable by inclusion of these new or re-purposed agents in the regimens [10]. Similarly, A study in South Africa demonstrates that bedaquiline (BDQ) is a safe drug and is associated with the high success rate for the MDR-TB and XDR-TB cohort even in high HIV burden areas [11, 12]. Recently, the WHO released the updated guidelines on the MDR-TB treatment on the basis of new experimental and observational evidence, in which the late-generation fluoroquinolones (FQs, i.e., levofloxacin and moxifloxacin), linezolid and bedaquiline are classified as preferred Group A drugs [13]. Subsequently, the definition of extensively drug-resistant TB (XDR-TB) has been revised by the WHO Global TB Programme, aiming to enable access to more effective treatment options for patients afflicted with drug-resistant strains [14]. The updated XDR-TB is defined as infection with an MDR-TB strain that is also resistant to any fluoroquinolone and at least one additional Group A drug. This redefinition points to increasing progression of the severity of the disease, and predicts poor clinical outcomes. The appropriate drug susceptibility testing (DST) methods are required to optimize the use of Group A drugs to improve the treatment of MDR-TB. Unfortunately, the commercial DST methods remain limited to assess in vitro susceptibility of MTB isolates to bedaquiline and linezolid. The national surveillance of anti-TB resistance in MDR-TB is thus essential to assist countries in planning the scale-up of patient management.

Despite a significant achievement in tackling the TB epidemic over the past years, China has a serious MDR-TB burden, with an estimated 74% rifampicin-resistant/MDR-TB cases in 2019 [1, 15]. Findings on temporal surveillance demonstrated that the prevalence of MDR-TB has dramatically increased in China [16]. The spread of MDR- and XDR-TB has been a major threat from both a clinical and a public health perspective. Although recent changes in the WHO recommendations for treatment of RR/MDR-TB patients have replaced injectables with bedaquiline in preferred therapy [17], the treatment of these patients majorly relies on long-course regimens stipulated by the WHO due to limited accessibility to bedaquiline. Only 54% of RR/MDR-TB cases who started treatment achieved a favorable outcome, which was recognized as a threat to TB control efforts in this country. To date, we still lack national data regarding the prevalence of XDR-TB in China. To address this concern, we conducted a study to determine in vitro susceptibilities of MTB isolates to FQs, bedaquiline and linezolid in a Chinese MDR-TB cohort. The putative drug target genes conferring drug resistance were screened by DNA sequencing.

## Materials and methods

### Bacterial isolates

Between February 2018 and June 2019, a retrospective cohort study was conducted by inclusion of MDR-TB patients in 13 hospitals, aiming to determine clinical efficacy of MDR-TB patients treated with BDQ-containing regimens [18]. The baseline MTB isolates were used for in vitro drug susceptibility testing. The positive cultures were stored in 7H9 medium supplemented with 10% of oleic acid-albumin-dextrose-catalase (OADC) and 5% glycerol. prior to determining minimum inhibitory concentration (MIC) values, the isolates were recovered on Löwenstein–Jensen (L–J) medium for 4 weeks at 37 °C.

### Minimum inhibitory concentration

The MICs of MTB isolates to antimicrobial agents were assessed with Middlebrook 7H9 broth microdilution using the Thermo Fisher frozen microtiter plates as previously reported [19]. Briefly, the 4-week-old fresh colonies were scraped from the surface of L–J slants. Followed by vigorously votexing for 1 min, a suspension of MTB isolate was adjusted to the turbidity of a 1.0 McFarland standard. The suspension was diluted to 1:20 as inoculum. Then 100 μL of this inoculum was automatically added into each well of the 96-well drug-containing plate. Plates were sealed and incubated at 37 °C incubator for 10 days, 14 days or 21 days depending on visible growth in control well. The Sensititre Vizion sytem (Thermo Fisher Scientific, West Sussex, UK) was used to monitor the growth of mycobacteria in each well, and the MICs were automatically interpretated with SWIN® software (Thermo Fisher Scientific, West Sussex, UK). The concentrations of anti-TB drugs tested were as follows: ofloxacin (OFX, 0.12–8 μg/mL), levofloxacin (LFX, 0.12–4 μg/mL), moxifloxacin (MFX, 0.06–4 μg/mL), bedaquiline (0.008–4 μg/mL), and linezolid (0.12–8 μg/mL). The MIC was defined as the lowest drug concentration inhibiting the visual growth of mycobacteria. MTB H37Rv (ATCC 27,249) was tested in each batch as a quality control strain. The MIC breakpoint concentrations were defined as 2 μg/mL for OFX and LFX, 0.5 μg/ml for MFX, 0.25 μg/ml for BDQ, and 1.0 μg/ml for LZD, respectively.

### DNA extraction and sequencing

Genomic DNA of fresh MTB colonies was extracted with MasterPure™ Complete DNA&RNA Purification kit (Lucigen corporation, Wisconsin, USA) according to the manufacturer’s instruction. The partial fragments of genes conferring drug resistance were amplified for DNA sequencing, including *gyrA* and *gyrB* for FQs, *atpE*, *Rv0678*, *Rv1979c* and *pepQ* for BDQ, and *23S rRNA* and *rplC* for LZD, respectively. The genomic DNA was used as template to perform PCR reaction as follows: 25 μL of 2xPCR mixture (CWBiotech, Beijing, China), 0.2 μM of each primer and 1 μL of template DNA. PCR program was done as previously reported [19–21]. The primer pairs used herein are listed in Additional file 1: Table S1. PCR products were sent to the RuiBiotech Company for DNA sequencing service (RuiBiotech, Beijing, China). The sequences were aligned with the corresponding genes of the reference MTB strain H37Rv using multiple sequence alignments at the National Center for Biotechnology Information (NCBI) website.

### Definitions

MDR-TB was defined as patients infected with MTB strain resistant to at least rifampicin and isoniazid. Pre-XDR was defined as patients infected with MDR-TB plus additional resistance to any fluroquinolone. XDR-TB was defined as patients infected with MDR-TB plus resistance to both FQ and at least one additional Group A drug [14].

## Results

### Drug susceptibility profiles of MDR-TB isolates

**Fig. 1:**
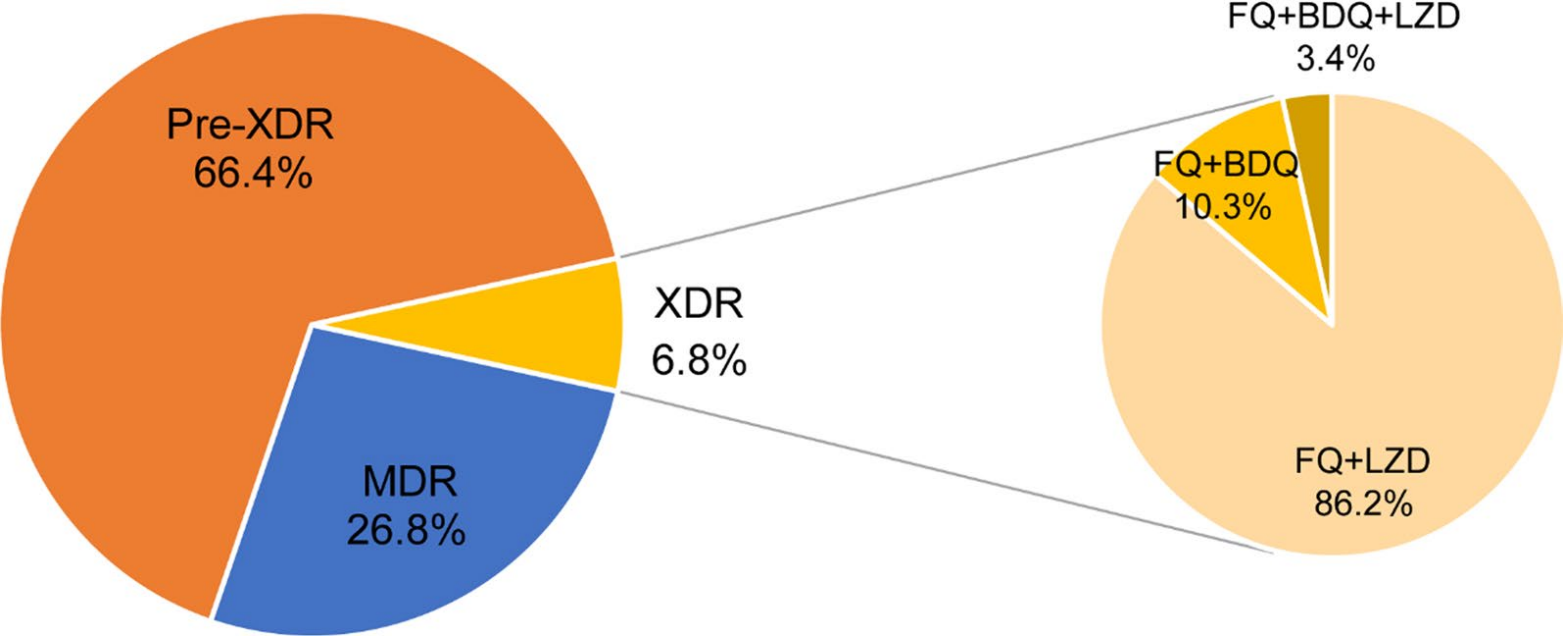
Distribution of MDR-TB isolates stratified to in vitro susceptibility testing. MDR, multidrug resistance; pre-XDR, pre-extensive drug-resistance; XDR, extensive drug-resistance; FQ, fluroquinolone; LZD, linezolid; BDQ, bedaquiline

A total of 425 MDR-TB isolates were included in our analysis. Table 1 shows the drug susceptibility profiles of these isolates, of which, 311 (73.2%) were resistant to FQ and 171 (40.2%) to any second-line injectable drug (SLID). In addition, 143 (33.6%) had multiresistance to both FQ and SLID. We further evaluated in vitro susceptibility of these isolates with broth microdilution method. LZD and BDQ resistance were noted in 30 (7.1%) and 10 (2.4%) isolates. On the basis of latest definitions, 114 (26.8%) were MDR-TB, 282 (66.4%) were pre-XDR-TB, and 29 (6.8%) were XDR-TB. Among 29 XDR-TB isolates, 25 (86.2%) were resistant to LZD, 3 (10.4%) to BDQ, and 1 (3.4%) to both LZD and BDQ (Fig. 1). Of note, resistance to LZD and BDQ was also recorded in MDR-TB group, consisting of 4 LZD-resistant and 6 BDQ-resistant isolates, respectively.

**Table 1:** Drug susceptibility profiles of MDR-TB isolates enrolled in this study

Drug susceptibility profile	No. of isolates	Proportion (%)
INH + RIF	86	20.2
INH + RIF + FQ	168	39.5
INH + RIF + SLID	28	6.6
INH + RIF + FQ + SLID	143	33.6
Total	425	100.0

INH, isoniazid; RIF, rifampicin; FQ, fluoroquinolone; SLID, second-line injectable drugs

### Mutations in the quinolone resistance-determining regions (QRDRs) of gyrA and gyrB genes

Mutations conferring FQ resistance were investigated by Sanger sequencing. As summarized in Table 2, among 311 FQ-resistant isolates, 265 (85.3%) harbored genetic mutations within QRDRs. The most common mutations were observed at codon 94 of *gyrA*, where the wild-type serine was replaced with six different amino acid: Asp94Ala (*n* = 17), Asp94Asn (*n* = 19), Asp94Gly (*n* = 96), Asp94His (*n* = 4), Asp94Tyr (*n* = 3) and Asp94Val (*n* = 8), accounting for 47.3% (*n* = 147) of FQ-resistant MTB isolates. The second most prevalent mutation was observed in codon 90, resulting in the amnio acids substitution of Ala to Val (*n* = 76, 24.4%). For the *gyrB* gene, synonymous mutations were found in 7 (2.3%) FQ-resistant isolates. The correlation between genetic mutations and MICs was further analyzed. Interestingly, the substitutions of Ala90Val and Ser91Pro within gyrA were associated with low-level FQ-resistance, and 27.6% (*n* = 21) and 36.4% (*n* = 12) of isolates tested were susceptible to MFX, respectively. In contrast, the isolates with the mutations at codon 94 had higher MIC values, which were all resistant to MFX.

**Table 2:** Mutations in the quinolone resistance-determining region (QRDR) of *gyrA* and *gyrB* and fluoroquinolone MICs

Mutations in QRDR	N (%)	FQ	No. of isolates with corresponding MIC (mg/L)						
			0.12	0.25	0.5	1	2	4	8
GyrA Asp89Asn	2 (0.6)	MFX	0	1	1	0	0	0	0
		OFX	0	0	0	0	0	2	0
		LFX	0	0	0	0	1	1	0
GyrA Ala90Val	76 (24.4)	MFX	18	3	16	23	8	8	0
		OFX	0	0	0	0	17	38	21
		LFX	0	0	0	0	56	20	0
GyrA Ser91Pro	33 (10.6)	MFX	3	9	10	4	7	0	0
		OFX	0	0	0	3	7	19	4
		LFX	0	0	0	4	25	4	0
GyrA Asp94Ala	17 (5.5)	MFX	0	0	10	4	3	0	0
		OFX	0	0	0	0	2	12	3
		LFX	0	0	0	0	15	2	0
GyrA Asp94Asn	19 (6.1)	MFX	0	0	4	4	7	4	0
		OFX	0	0	0	0	3	4	12
		LFX	0	0	0	0	4	15	0
GyrA Asp94Gly	96 (30.9)	MFX	0	0	10	14	44	28	0
		OFX	0	0	0	0	3	9	84
		LFX	0	0	0	0	11	85	0
GyrA Asp94His	4 (1.3)	MFX	0	0	0	0	0	4	0
		OFX	0	0	0	0	0	0	4
		LFX	0	0	0	0	0	4	0
GyrA Asp94Tyr	3 (1.0)	MFX	0	0	0	0	3	0	0
		OFX	0	0	0	0	0	0	3
		LFX	0	0	0	0	0	3	0
GyrA Asp94Val	8 (2.6)	MFX	0	0	0	2	4	2	0
		OFX	0	0	0	0	2	4	2
		LFX	0	0	0	0	4	4	0
GyrB Ser447Phe	2 (0.6)	MFX	2	0	0	0	0	0	0
		OFX	0	0	0	0	0	2	0
		LFX	0	0	0	0	2	0	0
GyrB Asp461Asn	1 (0.3)	MFX	0	0	0	0	1	0	0
		OFX	0	0	0	0	0	0	1
		LFX	0	0	0	0	0	1	0
GyrB Asp500Asn	1 (0.3)	MFX	0	0	1	0	0	0	0
		OFX	0	0	0	0	0	1	0
		LFX	0	0	0	0	1	0	0
GyrB Ala504Thr	3 (1.0)	MFX	0	0	0	0	0	3	0
		OFX	0	0	0	0	0	0	3
		LFX	0	0	0	0	0	3	0
WT	46 (14.7)	MFX	0	7	15	12	8	4	0
		OFX	0	0	0	1	15	21	9
		LFX	0	0	1	3	24	16	2
Total	311 (100.0)	MFX	23	20	67	63	85	53	0
		OFX	0	0	0	4	49	112	146
		LFX	0	0	1	7	143	158	2

MIC, minimum inhibitory concentration; WT, wild-type; FQ, fluoroquinolone; MFX, moxifloxacin; OFX, ofloxacin; LFX, levofloxacin

### Mutations conferring BDQ and LZD resistance

We analysed the mutation within genes conferring BDQ resistance. Only mutations within the *Rv06*78 gene were found to confer BDQ resistance in our cohort. Of 10 BDQ-resistant isolates, 4 (40.0%) had Rv0678 mutations, while no mutations were noted in the other 6 isolates (Table 3). Notably, all BDQ-resistant isolates had MICs bordering the susceptible breakpoint, varying from 0.25 to 0.5 μg/ml.

**Table 3:** Profiling of genetic mutations and MICs for BDQ-resistant MTB isolates

Locus	Mutation	No. of isolates with corresponding MIC (μg/ml)			Total (%)
		0.25	0.5	1	
*Rv0678*	Ala86Val		1		1 (10.0)
	Ile108Thr		1		1 (10.0)
	Arg109Pro	1			1 (10.0)
	Deletion A in codon 110		1		1 (10.0)
Wild-type		4	1	1	6 (60.0)
Total		5	4	1	10 (100.0)

MIC, minimum inhibitory concentration; BDQ, bedaquiline; MTB, *Mycobacterium tuberculosis*

For LZD resistance, 53.3% of LZD-resistant isolates carried genetic mutations in *rplC* or 23S rRNA. The most frequent mutation was Cys154Arg (*n* = 12) in the *rplC* gene, which revealed MICs ranging from 4 to > 8 μg/ml. In addition, two types of nucleotide substitutions in 23S rRNA were identified among 4 LZD-resistant isolates, including 3 isolates with G2270T and 1 isolate with G2270C. The MICs of isolates carrying these mutations were from 4 to 8 μg/ml (Table 4).

**Table 4:** Profiling of genetic mutations and MICs for LZD-resistant MTB isolates

Locus	Mutation	No. of isolates with corresponding MIC (μg/ml)				Total (%)
		2	4	8	> 8	
*rplC*	Cys154Arg		5	5	2	12 (40.0)
23S rRNA	G2270C		1			1 (3.3)
	G2270T		2	1		3 (10.0)
Wild-type		7	6	1		14 (46.7)
Total		7	14	7	2	30 (100.0)

MIC, minimum inhibitory concentration; LZD, linezolid; MTB, *Mycobacterium tuberculosis*

### Clinical outcomes of MDR-TB with resistance to both BDQ and LZD

In our cohort, we recorded two MDR-TB patients with resistance to both BDQ and LZD. We thus assessed the clinical outcomes of these MDR-TB patients by the administration of BDQ-containing regimens. As shown in Table 5, out of these patients, only one patient achieved successful outcome; whereas the other patient experienced continuous positive culture of MTB despite inclusion of efficacious MFX.

**Table 5:** Clinical outcomes of patients infected with resistance to both BDQ and LZD

Patient ID	Patient category	Drug susceptibility	Mutation conferring BDQ resistance	Mutation conferring LZD resistance	Treatment regimen	Sputum culture conversion	Clinical outcome
040,012	Previously treated	MDR	WT	Cys154Arg (*rplC*)	Bdq/Lzd/Mfx/Cs/Am/Pto	8 weeks	Cured
010,121	Previously treated	XDR	Ile108Thr (*Rv0678*)	WT	Bdq/Lzd/Mfx/Cs/Cfz/Am	NA	Treatment failure

MDR, multidrug-resistance; XDR, extensive drug-resistance; BDQ, bedaquiline; LZD, linezolid; Mfx, moxifloxacin; Cs, cycloserine; Am, amikacin; Pto, protionamide; Cfz, clofazimine; NA, not available; WT, wild-type

## Discussion

Drug resistance surveillance is of importance to identify and predict the impact of new empirical anti-TB drug prescribing [22]. To our best knowledge, this was the first snapshot of the prevalence of XDR-TB among MDR-TB patients in a high-burden setting. Our data demonstrate that the rate of XDR-TB was noted in 6.8% of patients afflicted with MDR-TB, which was significantly decreased from 33.6% on the basis of previous definition. This low rate of XDR-TB was majorly attributed to relatively late introduction of BDQ and LZD for clinical management of MDR-TB in China, thereby preventing accumulation of drug resistant mutations [18]. Consequently, this holds great promise for MDR-TB treatment with WHO-endorsed regimens containing BDQ-LZD combination.

Although the overall prevalence of XDR-TB was low, the high prevalence of FQ-resistance in MDR-TB patients warrants national attention. In a recent population-based study in China, the prevalence of MFX resistance was markedly increased from 3.0% in 2000 to 7.7% in 2010 [23]. Similar results were observed by surveillance data in Beijing, which demonstrated that a statistically significant increase in LFX resistance over the past decade [20]. This phenomenon is probably the result of overuse of FQs in the treatment of undiagnosed bacterial infections in China in view of their promising efficacy and low occurrence of adverse events. In addition, widespread use of FQs in animal and food industries has resulted in reported excessive accumulation of antibiotics in environmental water samples in China [24]. The unexpected exposure to environmental FQs is another possible explanation for increasing FQ resistance in our MDR-TB cohort. Thus our primary results directly address the concern that essential interventions are required to reduce the misuse of antibiotics in clinical practice, as well as in livestock and food industries.

The acquisition of drug resistance is associated with the presence of genetic mutations conferring resistance [25]. The most frequently observed mutations in BDQ-resistant isolates were reported in *Rv0678*, a transcriptional repressor of efflux genes participating in the regulation of expression of MmpS5–MmpL5 [25, 26]. Consistent with previous investigation, mutations in *Rv0678* were the major mechanism for BDQ resistance in our study. The high prevalence of *Rv0678* mutation may be related to the prior exposure to clofazimine among MDR-TB patients considering the cross-resistance between these two drugs. Of note, mutations within the *Rv0678* are highly diverse, with 4 unique mutations at 4 different positions. Although the basis for the highly diverse *Rv0678* mutations remains unclear, our results reveal that the DNA sequencing is more suitable for identify mutations within *Rv0678* locus rather than conventional PCR-based assays, such as real-time PCR and line probe assay. We also found that approximate half of BDQ-resistant isolates harbored no mutations within other known BDQ resistance genes. Similar results were noted in MTB isolates with LZD resistance. In addition to target gene mutations, multiple mechanisms confer drug resistance, such as cell wall permeability and efflux [27]. An experimental study by Velayati and co-workers found that the XDR-TB MTB isolates had thicker cell wall than MDR and susceptible isolates though viewing the ultrastructure of cell wall [28]. Thus we hypothesize that the cell permeability-associated mechanism may play an important role of in these MTB isolates. The poor correlation between genetic mutations and phenotypic resistance forebodes unsatisfactory performance using molecular panel to diagnose resistance to these novel drugs. Further analysis is required to elucidate novel mechanisms conferring BDQ and LZD resistance, thereby boosting the development of rapid molecular diagnostics for drug resistance.

Despite limited data, we observed that a patient with resistance to both LZD and BDQ but susceptible to MFX experienced treatment failure. In a recent BDQ clinical trial, the administration of BDQ-containing regimens provides particular benefit for pre-XDR- and XDR-TB patients [18]. On one hand, the existing evidence from studies confirmed BDQ as a cornerstone in the treatment of MDR-TB patients. Although the revised definitions of XDR-TB have emphasized the importance of BDQ, it excludes the patients at high risk of treatment failure from pre-XDR and XDR-TB group. More clinical data is required to subtly stratify drug-resistant TB patients according to their risk of unfavorable outcomes. On the other hand, there is an urgent need for commercial laboratory tests of the sensitivity of tubercle bacilli to BDQ and LZD, such as phenotypic DST using MGIT, which is essential to guide the choice of chemotherapy for MDR-TB patients.

We also acknowledged several obvious limitations to our study. First, despite the enrollment of MDR-TB isolates in our cohort, the relatively small number of MTB isolates tested may result in potential sampling bias. Second, the patients were non-responsive MDR-TB, possibly leading to overestimation of XDR-TB in Chinese population. Third, the MIC values were not assessed in triplicate. Hence, the lack of repeated experiments may introduce the systematic error by microdilution method, especially for isolates with MICs close to the breakpoint. Finally, whole genome sequencing (WGS) was not conducted to identify the molecular mechanism conferring BDQ and LZD resistance among MTB isolates without known mutations. Specially, a proportion of drug-resistant isolates may be caused by the presence of heteroresistant subpopulation, which were missed by Sanger Sequencing. Further WGS analysis is of importance to elucidate the molecular mechanism underlying resistance to these agents.

In conclusion, our results demonstrate that initial XDR-TB occurs in 6.8% of MDR-TB patients in our Chinese population, whereas the high prevalence of FQ-resistance in MDR-TB patients warrants national attention. Approximately half of BDQ-resistant and LZD-resistant isolates harbor no mutations within known resistance genes. The low prevalence of XDR-TB holds great promise for MDR-TB treatment with WHO-endorsed regimens containing BDQ-LZD combination.

## Supplementary Information

**Additional file 1**. Primers used in this study for PCR amplification and sequencing.

## Data Availability

The datasets in the present study are accessible from the corresponding author on reasonable request.

## Abbreviations

MTB: *Mycobacterium tuberculosis*
XDR-TB: Extensively drug-resistant TB
MDR-TB: Multidrug-resistant TB
FQs: Fluoroquinolones
BDQ: Bedaquiline
INH: Isoniazid
RIF: Rifampicin
SLID: Second-line injectable drugs
MIC: Minimum inhibitory concentration
WT: Wild-type
MFX: Moxifloxacin
OFX: Ofloxacin
LFX: Levofloxacin
LZD: Linezolid
Cfz: Clofazimine
QRDRs: Quinolone resistance determining regions
