# Supplementary material for: Prevalence of extensively drug-resistant tuberculosis in a Chinese multidrug-resistant TB cohort after redefinition
Source: Antimicrob Resist Infect Control. 2021 Aug 26;10:126. doi: 10.1186/s13756-021-00995-8 (PMC8393791; doi:10.1186/s13756-021-00995-8)
Supplement: Supplementary file 1 — Additional file 1. Primers used in this study for PCR amplification and sequencing. [file 13756_2021_995_MOESM1_ESM.docx]

**Supplemental Table 1** Primers used in this study for PCR amplification and sequencing

| **Gene** | **Length**  **(bp)** | **Primer** |
| --- | --- | --- |
| *gyrA* | 398 | 5’-GATGACAGACACGACGTTGC-3’F  5’-GGGCTTCGGTGTACCTCAT-3’R |
| *gyrB* | 428 | 5’-CCACCGACATCGGTGGATT-3’F  5’-CTGCCACTTGAGTTTGTACA-3’R |
| *atpE* | 146 | 5’-TAGACATCGCGTGCGGATTGGT -3’ F |
|  |  | 5’-GCGTGGTCAACAACGTGGCAAT -3’ R |
| *Rv0678* | 232 | 5’-TCGCCAGCAACTTAGGGCAATACA -3’ F |
|  |  | 5’-TCCGATGACGTAGCCGCAAACTAG -3’ R |
| *pepQ* | 171 | 5’-GTTTGGTGCCGCTCAACTCGTATC -3’ F |
|  |  | 5’-GGTACGGCGCATACGACGCAGATA -3’ R |
| *Rv1979c* | 225 | 5’-CGCCCTACGGGAAACCAACAAAGA -3’ F |
| *23S rRNA*  *rplC* | 494  594  643  513  390  419 | 5’-GCGGAACAAGTGGAACGGCACGAC -3’R  P1 5’- AGAACCTTGCCCGCCGAAAGAC-3’F  P2 5’-CCGAAGTTACGGGGGCATTTTG -3’R  P3 5’- GGTTGAAGACTGAGGGGATGAG-3’F  P4 5’-GCCTTAGGTCCCGACTCACCCT -3’R  P5 5’- CCCAAACCGACACAGGTGGTCA-3’F  P6 5’- AAACTACCCGCCAGGCACTGTC -3’R  P7 5’-GGGACAGTGCCTGGCGGGTAGT-3’F  P8 5’-AGATGCTTTCAGCGGTTATCCT -3’R  P9 5’-CCTCGCTGCCCAGAA AGGGA-3’F  P10 5’-CATCGGCGCTGGCAGGCTTAG -3’R  5’-GCTGCGGCTGGACGACTC-3’F  5’-CTCTTGCGCAGCCATCACTTC-3’R |
